# Phylogenetic position of *Bopyroides hippolytes*, with comments on the rearrangement of the mitochondrial genome in isopods (Isopoda: Epicaridea: Bopyridae)

**DOI:** 10.1186/s12864-022-08513-9

**Published:** 2022-04-02

**Authors:** Ruiwen Wu, Rongxiu Guo, Qianqian Xi, Gustav Paulay, Jianmei An

**Affiliations:** 1 School of Life Science, Shanxi Normal University, Taiyuan, 030031 P. R. China; 2 Florida Museum of Natural History, University of Florida, Gainesville, FL 32611-7800 USA

**Keywords:** Parasitic isopods, Phylogenetic relationship, Mitogenomes, Gene arrangement

## Abstract

**Background:**

Classification of parasitic bopyrids has traditionally been based on morphological characteristics, but phylogenetic relationships have remained elusive due to limited information provided by morphological data and tendency for loss of morphological features as a result of parasitic lifestyle. Subfamily Argeiinae was separated from Bopyrinae based on morphological evidence, although the assignment of all genera has not been phylogenetically evaluated. *Bopyroides hippolytes* has been traditionally classified in Bopyrinae, but divergent morphological characters make this assignment questionable. To investigate the relationship of bopyrines, we sequenced the complete mitochondrial genome of *B. hippolytes* and four mitochondrial genes of two other Bopyrinae.

**Results:**

The phylogenetic trees based on separate and combined *cox1*and 18S sequence data recovered Bopyridae as robustly monophyletic, but Bopyrinae as polyphyletic. *Bopyroides hippolytes* was a close sister to *Argeia pugettensis,* type species to Argeiinae. Mitochondrial phylogenomics also suggested that *B. hippolytes* was close to Argeiinae. We also found a novel gene order in *B. hippolytes* compared to other isopods.

**Conclusions:**

*Bopyroides hippolytes* should be excluded from the Bopyrinae and has a close affinity with *Argeia pugettensis* based on molecular and morphological data. The conserved syntenic blocks of mitochondrial gene order have distinctive characteristics at a subordinal level and may be helpful for understanding the higher taxonomic level relationships of Isopoda.

**Supplementary Information:**

The online version contains supplementary material available at 10.1186/s12864-022-08513-9.

## Introduction

Bopyridae Rafinesque, 1815 is a parasitic family of isopod crustaceans, with 10 subfamilies, 208 genera and 634 species (Boyko et al., 2008 onwards) [1]. Bopyrids stunt the growth and reduce the reproductive abilities of their hosts [2]. Molecular phylogenetic research on Bopyridae has been scarce, partly because of the challenge of assembling sequenceable material. Molecular phylogenetic analyses began only recently. Boyko et al. [3] assessed phylogenetic relationships among Bopyridea Rafinesque, 1815 and Cryptoniscoidea Kossmann, 1880 using 18S sequence data, and recovered Bopyridae as monophyletic. Yu et al. [4] and An et al. [5] sequenced and analyzed the mitochondrial genome of the bopyrids *Gyge ovalis* (Shiino, 1939) and *Argeia pugettensis* Dana, 1853, respectively, but the paucity of comparative sequence data prevented resolution of bopyrid relationships at the subfamily level.

With some exceptions, members of each bopyrid subfamily are restricted to hosts from one decapod infraorder [6]. Bopyrinae, Argeiinae and Pseudioninae infest the branchial chambers of caridean shrimp. Shiino [7] grouped a series of bopyrid genera, including *Argeia* Dana, 1853, *Stegoalpheon* Chopra, 1923, *Bopyrosa* Nierstrasz & Brender à Brandis, 1923, *Parargeia* Hansen, 1897 into his *Bopyrus*-group, later recognized as the Bopyrinae. Markham [8] placed these four genera together with *Argeiopsis*, into his newly erected subfamily Argeiinae. He noted that Argeiinae can be distinguished from Bopyrinae in that the female of the latter has a head that is not oval or fusiform and is usually fused with the pereon, an oval or deltoid body outline, some or all pleomeres fused at least on one side, lateral plates and uropods that are greatly reduced or absent, and pleopods that are generally biramous.

*Bopyroides* Stimpson, 1864 is currently placed in the Bopyrinae but does not fit well there. Females have an oval head, distinctly separated from pereon, all pleomeres are distinct without any fusion, have prominent dorsolateral bosses and tergal projections, and pleopods are reduced or uniramous tuberculiform [9–11]. Thus, there remains uncertainty about the boundaries between the Bopyrinae and Argeiinae, and the validity of Argeiinae has also been questioned [12].

*Bopyroides* includes three valid species, all carid parasites in Palearctic waters. *Bopyroides hippolytes* (Kröyer, 1838) parasitizes several shrimp species in the families Thoridae and Pandalidae in the cold, northern waters of the Pacific, Atlantic, and Arctic Oceans [5, 13], and has an unusually great range among Bopyridae. Kröyer [10] described this species in *Bopyrus*, infesting *Lebbeus olaris* (Sabine, 1824) in Greenland. Bate & Westwood [14] moved the species to *Gyge*, then Sars [15] transferred it to *Bopyroides*, established by Stimpson [16] for *Bopyroides acutimarginatus* Stimpson, 1864. Bonnier [17] described *Bopyroides sarsi* infesting *L. olaris* from the Arctic Ocean. Richardson [18] regarded *B. acutimarginatus* and *B. sarsi* Bonnier, 1900 as synonyms of *B. hippolytes*. These synonymies and the placement of *Bopyroides* in Bopyrinae have been accepted by subsequent authors [6, 13, 19–23], even though several characters conflict with that subfamilial assignment as noted above. Scott [9] described *Pleuroctypta cluthae* infesting *Pandalina brevirostris* (Rathke, 1843) (Pandalidae) from Clyde Sea (Scotland). Bourdon [21] transferred it to *Bopyroides* and compared it with *B. hippolytes* in detail. Rybakov& Avdeev [11] described *B. shiinoi* from the Russian Pacific, based on a female with five pairs of uniramous pleopods. Boyko [23] suggested that Shiino’s [19] and Kim & Kwon’s [22] specimens of *B. hippolytes* from Japan and Korea are also referable to *B. shiinoi*, as all have female with five pairs of uniramous pleopods. Classification history and synonym for the three *Bopyroides* species are shown in Table 1.

**Table 1:** Classification history and synonym for the three *Bopyroides* species

**Kröyer, 1838** [10]	**Bate & Westwood, 1868** [14]	**Sars, 1898** [15]	**Richardson, 1905** [18]
*Bopyrus hippolytes* Kröyer, 1838 [10]	*Gyge hippolytes* (Kröyer, 1838) [10]	*Bopyroides hippolytes* (Kröyer, 1838) [10]	*Bopyroides hippolytes* (Kröyer, 1838) [10]
			*Bopyroides acutimarginatus* (Stimpson, 1864) [16] syn
			*Bopyroides sarsi* (Bonnier, 1900) [17] syn
**Scott, 1902** [9]	**Bourdon, 1968** [21]		
*Pleuroctypta cluthae* Scott, 1902 [9]	*Bopyroides cluthae* (Scott, 1902) [9]		
**Rybakov & Avdeev (1991)** [11]			
*Bopyroides shiinoi* Rybakov & Avdeev, 1991 [11]			

Key to species of *Bopyroides*:

1. Pleomere 6 of female with lateral, biramous extension…………………*B. cluthae*
  Pleomere 6 of female not laterally extended, round or truncate……………2
2. Female with four pairs of uniramous pleopods, head of male with straight posterior margin…*B. hippolytes*
  Female with five pairs of uniramous pleopods, head of male with curved posterior margin…*B. shiinoi*

We sequenced the mitogenome and 18S rRNA of three species currently assigned to the Bopyrinae: *Bopyroides hippolytes*, *Bopyrella malensis* Bourdon, 1980, and *Parabopyrella* cf. *mortenseni* (Bourdon, 1980), and analyzed their phylogenetic placements among bopyrids based on this and other available sequence data. We also compared gene arrangements across all available mitogenomes of the Isopoda and describe a novel mitochondrial gene order in bopyrids.

We addressed the following questions: (1) Is the Bopyrinae monophyletic? (2) What is the phylogenetic position of *Bopyroides hippolytes*? (3) How has mitochondrial gene rearrangement in the Isopoda?

## Results

### Sequence alignment and data partitions

Sequence information for the four datasets after alignment and trimming are shown in Table 2. The subset partitions and best-fit models from Partition Finder and Model Finder are presented in Table S1.

**Table 2:** Alignment length and sequence information of four datasets prior to and after treatment in Gblocksv0.91b based on nucleotides (first three) and amino acids (13PCG)

Datasets	Original length (bp)	Treatment length by Gblocks (bp)	Variable sites	Parsimony informative sites	Nucleotide diversity
*cox1*	1548	1494	851	639	0.287
18S	2416	1541	682	393	0.043
*cox1* + 18S	3595	3541	1697	855	0.212
13PCG	4256	3627	2910	2588	0.283

### Phylogenetic position of *B. hippolytes* and molecular phylogeny of Bopyridae

**Fig. 1:**
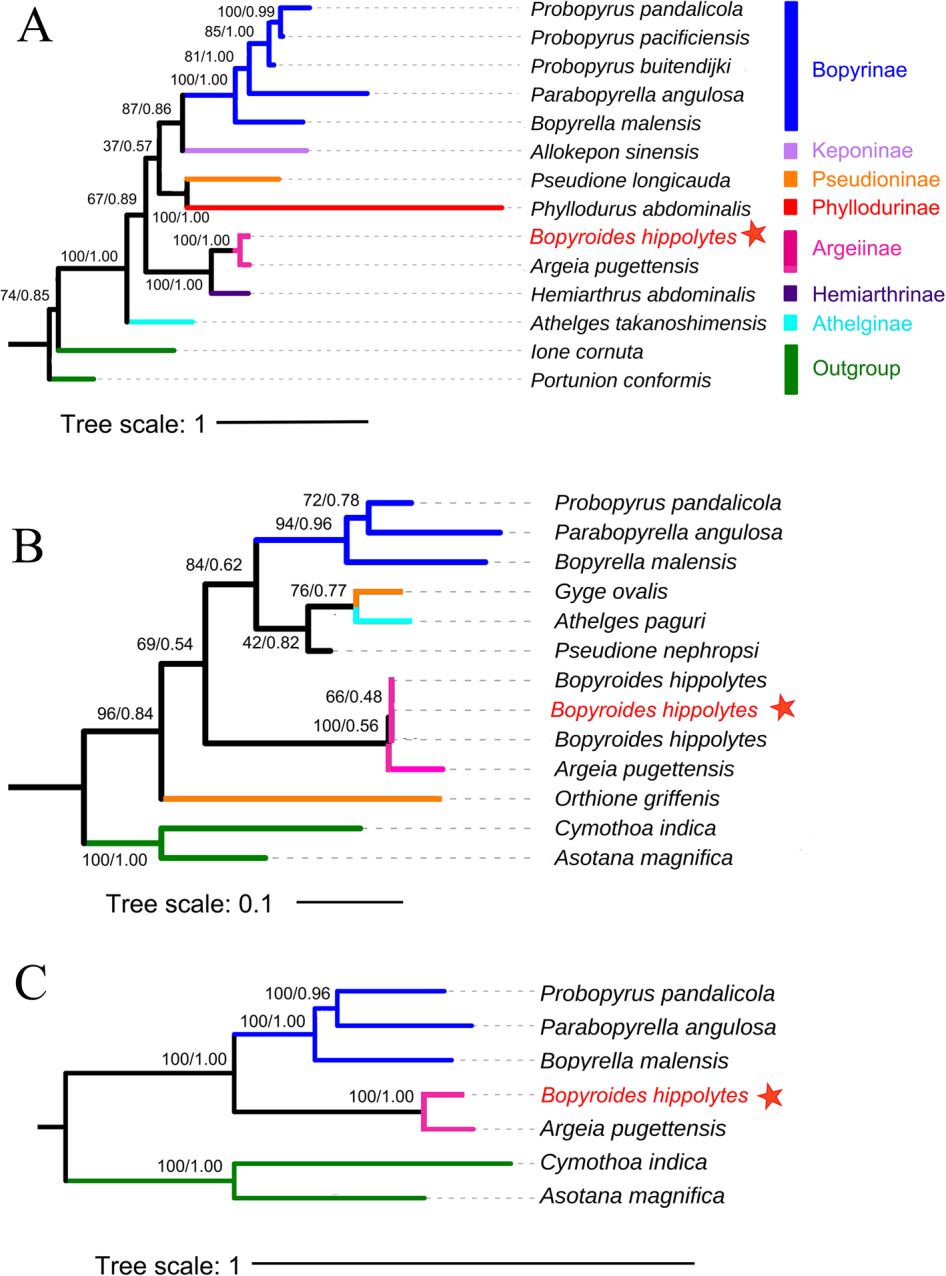
Phylogenetic trees based on (**A**) 18S, (**B**) *cox 1*, and (**C**) *cox1* and 18S. Numbers at nodes are statistical support values for ML (bootstrap support) / BI (Bayesian posterior probability). Bopyridae subfamilies showed on right and in color

The six phylogenetic trees obtained based on separate and combined *cox1* and 18S sequence data with ML and BI were congruent (Fig. 1). All analyses recovered most species currently assigned to the Bopyrinae (*Probopyrus pandalicola, P. pacificiensis, P. buitendijki, Parabopyrella mortenseni, Bopyrella malensis*) as robustly monophyletic, except for *Bopyroides hippolytes*, which was always a close sister to *Argeia pugettensis*. Our *Bopyroides hippolytes* sequence matches others in GenBank, further confirming the identification. The relationship of Argeiinae + Hemiarthrinae had strong support.

### Mitochondrial phylogenomics of Isopoda

**Fig. 2:**
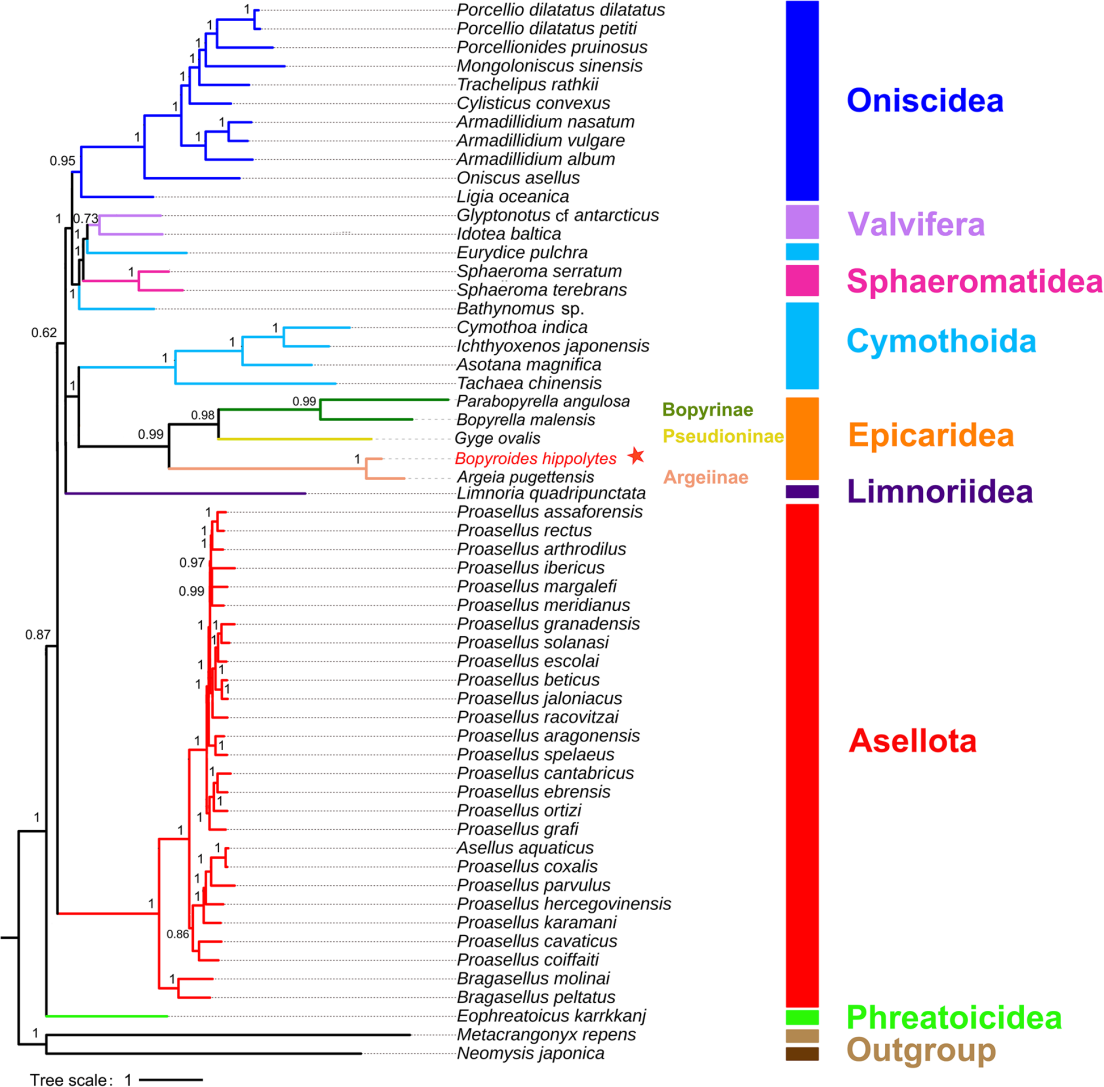
CAT-GTR phylogram based on nonpartitioned AA sequences of 13PCGs. Numbers at nodes are posterior probability values. Isopod suborders showed on right and in color. *Metacrangonyx repens* (Amphipoda) and *Neomysis japonica* (Mysida) are used as outgroups

Phylogenetic analysis based on AA sequences of the 13 PCGs recovered Phreatoicidea, then Asellota, as sister to all other sampled suborders in the Isopoda, albeit with modest support. All suborders except Cymothoida were monophyletic, although the polyphyly of Cymothoida also lacked robust support (Fig. 2). *Bopyroides hippolytes* was recovered as sister to *Argeia* (Argeiinae), distant from the other two Bopyrinae species.

### Novel mitochondrial gene arrangements

**Fig. 3:**
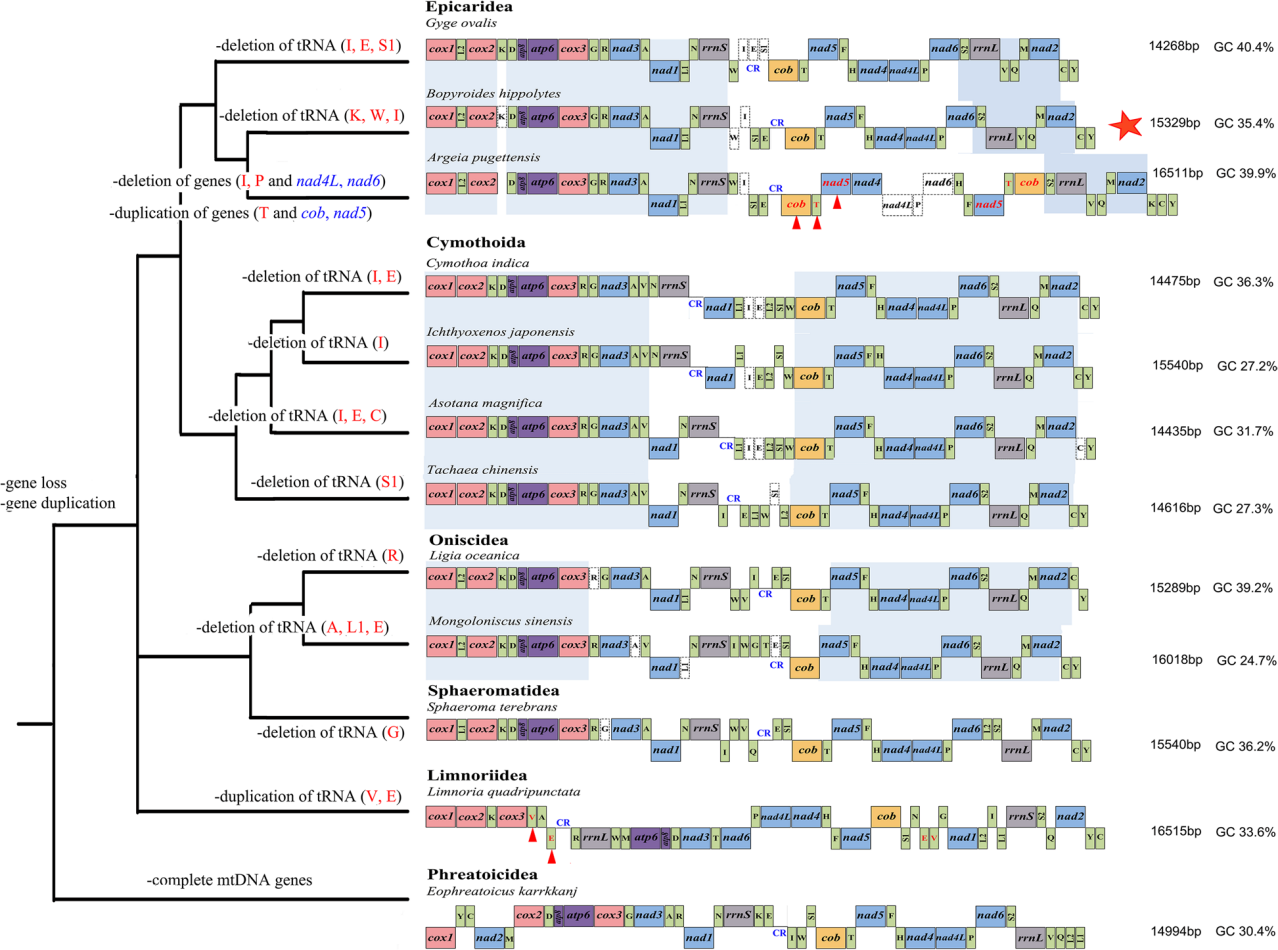
Comparison of gene arrangement of the complete mitogenomes for Isopoda mapped on a simplified isopod tree inferred from CAT-GTR. The most conserved syntenic blocks at the suborder level are marked with a blue background box. Gene duplications and deletions are marked with red triangles and white rectangles, respectively. The novel gene order in *B. hippolytes* is marked with the star. CR: control region

*Bopyroides hippolytes* has a unique mitochondrial gene order that differs from that of all other known in Isopoda (Fig. 3). The circular mitochondrial genome of *B. hippolytes* is 15,329 bp, with C + G content of 39.9%. It possesses the standard 13 PCGs and two rRNA genes (12S and 16S), but only 19 tRNA genes, as three tRNA genes (trnK, trnW and trnI) are missing compared to the standard animal mitogenome (Fig. 3). Nineteen genes are encoded on the +strand (light strand), whereas 4 protein-coding genes, 10 tRNAs and one rRNAs are located on the -strand (heavy strand). In base composition, *B. hippolytes* exhibits negative AT skew (− 0.129) and positive GC skew (0.039).

Isopods have undergone numerous gene duplication and deletion events in the mitogenome among taxa sampled to date, except for the basal Phreatoicidea, which retains a standard mitochondrial genome of 37 genes. While only tRNA genes were lost or duplicated in most isopod clades, PCGs were also duplicated in *Argeia pugettensis*. Expect *Limnoria quadripunctata*, the putative CR (control region) of all available isopod mitogenomes is located between the *rrnS* (+strand) and *cob* (−strand) genes.

## Discussion

### Phylogenetic relationships and taxonomic implications

Markham [8] separated the Argeiinae from the Bopyrinae based on several morphological features, including the shape of the head and pleopods, but considered these subfamilies to be closely related. *Bopyroides hippolytes* was originally described in *Bopyrus*, the type genus of Bopyrinae (Kröyer, 1838). While this widespread and common species has received substantial attention, and was transferred to its own genus, its subfamilial classification has not been discussed and remained accepted as Bopyrinae [6, 11, 13, 17–20, 22, 23].

The Bopyrinae was polyphyletic in all our phylogenetic analyses, with *Bopyroides hippolytes* separating from other bopyrine genera, but close to *Argeia pugettensis*. We suggest that *Bopyroides hippolytes* should be excluded from Bopyrinae and has a close affinity with *Argeia pugettensis*. This conclusion is also supported by some morphological data, such as the dorsolateral boss and tergal projection of *Bopyroides hippolytes*, described by Shiino [19], Allen [20], and Rybakov and Avdeev [11] that are characteristics of Argeiinae, not Bopyrinae. The two other species assigned to *Bopyroides*, *B. cluthae* and *B. shiinoi,* also have distinct pleomeres, prominent dorsolateral bosses and tergal projections [9, 11, 20] and likely are indeed congeneric. So, the boundary between Bopyrinae and Argeiinae is obscure, and the correct rank of *Bopyroides* need more data.

Our phylogenetic results are congruent with Boyko et al’s [3] analysis of bopyrids using 18S sequence data but extends it with greater sampling of Bopyrinae and Argeiinae. These two subfamilies are well separated on the phylogeny suggesting that their similarities perceived by Markham [24] are the result of convergence. There remain limited sequence data for this large family of parasitic isopods and further work will likely lead to additional changes in their classification.

### Gene rearrangement

Mitochondrial gene rearrangement provides useful information for understanding relationships at higher taxonomic levels [25, 26]. Duplication and deletion of tRNA are common in the rearrangement of mitochondrial genes in metazoans [27–29], likely caused by the replication slippage mechanisms [30, 31].

Most of the available isopod mitochondrial genomes in GenBank are partial or incomplete, with only 11 mitogenomes being complete (as of 30 Dec. 2020). We evaluated gene rearrangements by assembling the 12 available complete isopod mitogenomes, including *Bopyroides hippolytes* sequenced in this study. Among all complete isopod mitogenomes analyzed, tRNA deletions occur in all species except Limnoriidea and Phreatoicidea. These suborders are early cladogenesises among isopods and appear to retain the ancestral complement of tRNAs. Two general changes in the tRNA genes of all isopods include a reduction or loss of *trnI* and reduction of *trnC*. *trnI* was missing in both Epicaridea and Cymothoida (Fig. 3), and while it was retained in the other suborders, its cloverleaf structure was incomplete (loss of D-loop or TΨC). The D-loop region was lacking in the predicted secondary structures of *trnC* for all mitogenome analyzed. This phenomenon was also described by Kilpert and Podsiadlowski [25], who considered that this feature might be a putative autapomorphy of Isopoda. It is noticeable that an unusual deletion of *trnK* appears in *B. hippolytes*, reported for the first time in isopods. The area between *trnR* and *trnH* was considered a ‘hot spot’ of mitochondrial gene rearrangement in Isopoda [32, 33]. As the most parsimonious explanation of gene order change in this region, Kilpert and Podsiadlowski [25] assumed multiple translocation events. Because of the mixture of inversions and genome shuffling, tandem-duplication/random loss models were not a better way to explain the gene rearrangement [25, 34]. Crustacean taxa usually exhibit negative overall GC skews and positive AT skews on the heavy strand, but many studies have found that the bias for this strand is inversed in isopod mitogenomes [32–35]. *B. hippolytes* mitogenome in this study also exhibits negative AT skews and positive GC skews. It is considered to be the result of the architectural hypervariability and frequent inversions of the origin of mitochondrial replication (ORI) located in the control region (CR), where the changed replication order of two mitochondrial DNA strands consequently resulted in an inversed strand asymmetry [33, 35].

**Fig. 4:**
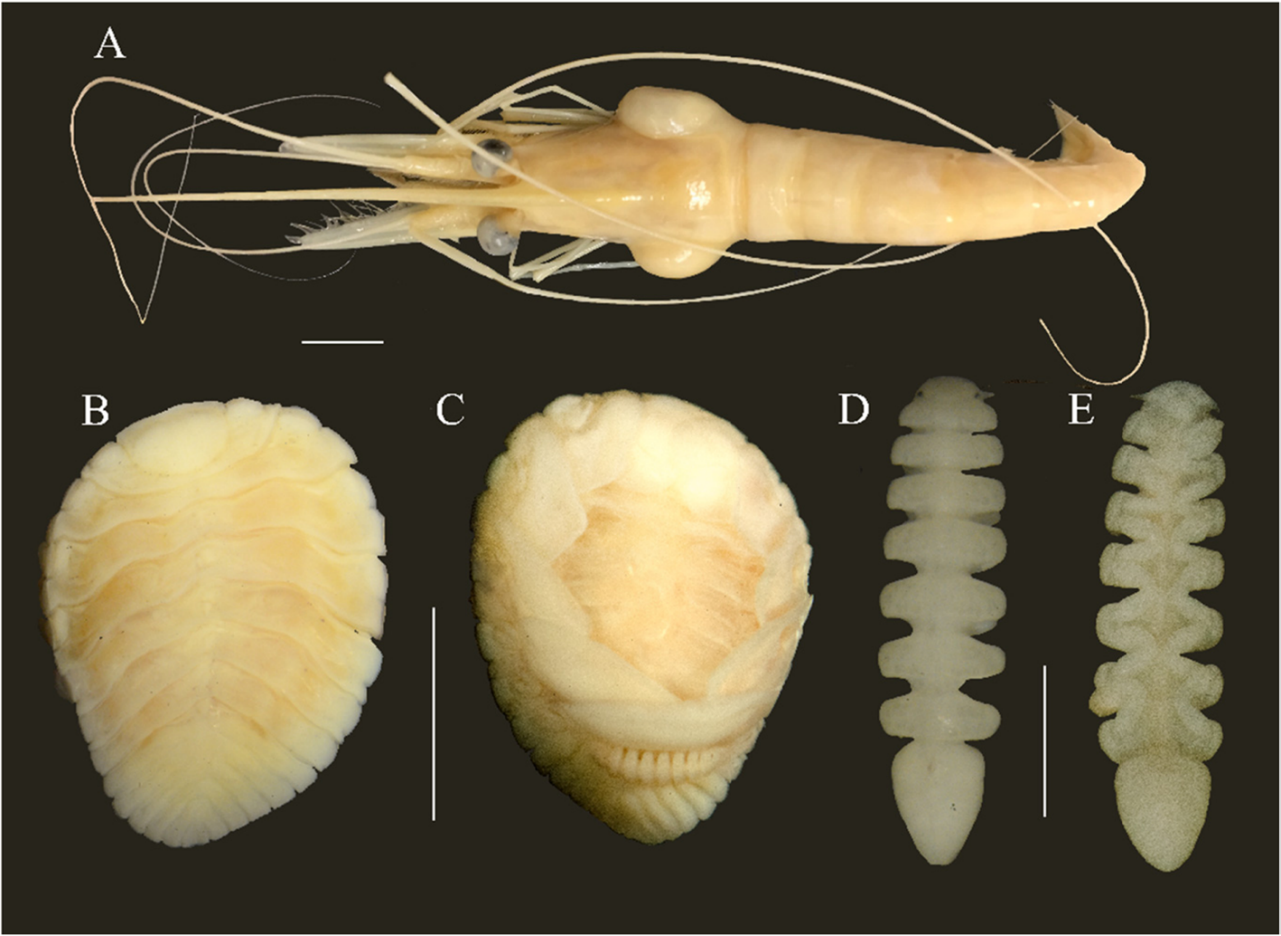
**A** Host *Pandalopsis dispar* Rathbun, 1902 (UF Arthropoda 45,427); dorsal (**B**) and ventral (**C**) view of female, and dorsal (**D**) and ventral (**E**) view of male voucher *Bopyroides hippolytes* (UF Arthropoda 45,428). Scale bars: **A** = 1 cm; **B** and **C** = 4 mm; **D** and **E** = 1 mm

Comparison of mitochondrial gene order across Isopoda shows relative stability within suborders, but substantial differences among suborders (Fig. 4). In Epicaridea, we speculated three small conserved syntenic blocks of the mitogenomes. Both deletion and duplication of genes occurred in this suborder, particularly *Argeia pugettensis*. Whereas in Cymothoida and Oniscidea, we supposed two large conserved blocks. Comparison of conserved regions of different suborders can indicate that mitogenomes gene order is an especially useful tool for higher taxonomy of isopod species. Sequencing of additional isopod mitogenomes promises to be a fertile ground for research.

## Conclusions

Our phylogenetic analyses based on *cox1*, 18S sequence and mitochondrial genome suggest that Bopyrinae is polyphyletic and *Bopyroides hippolytes* should be excluded from the Bopyrinae. We found a novel gene order in *B. hippolytes* compared to other isopods. The comparison of mitochondrial gene order shows that conserved syntenic blocks have distinctive characteristic at a subordinal level, and may be helpful for understanding the higher taxonomic level relationships of Isopoda.

## Materials and methods

### Taxon sampling

*Bopyroides hippolytes* (Fig. 4) (UF Arthropoda 45,428), infesting *Pandalopsis dispar* Rathbun, 1902 (UF Arthropoda 45,427), from the USA, Washington State, San Juan Islands, San Juan Channel, 80-120 m (48.578° N, 123.048° W, 17 Oct, 2015), collected by Gustav Paulay.

*Parabopyrella* cf. *mortenseni* (UF Arthropoda 44,587), infesting *Lysmata* sp. (UF Arthropoda 46,079), from Panama, Bocas del Toro Province, Cayo Hermanas, 3-3.5 m (9.268°, − 82.352°, 30 May 2016), collected by Matthieu Leray, Francois Michonneau and Robert Lasley. This specimen matches *Parabopyrella mortenseni*, described from Djibouti morphologically, but is unlikely to be conspecific given the great geographic separation.

Specimen of *Bopyrella malensis* (UF Arthropoda 46,906), infesting *Synalpheus* sp. (UF Arthropoda 46,905), from New Caledonia, Province Sud, Noumea lagoon, IIot St Marie, 1-10 m. (− 22.309°, 166.484°, 16 Nov, 2017), collected by Gustav Paulay, Daisuke Uyeno and Leonid Moroz.

Voucher specimens are deposited in the Florida Museum, University of Florida (UF).

Available 18S and *cox1* sequences of bopyrids were obtained from GenBank (Tables S2, S3).

### DNA extraction, amplification, sequencing, and annotation

Total genomic DNA was extracted from eggs or the pereon of female specimens using the genomic DNA rapid extraction kit (Aidlab Biotechnologies Co., Ltd) according to the manufacturer’s instructions. 18S rRNA gene region was amplified and sequenced using the same primers from Boyko et al. [3]. PCR conditions were: denaturation at 98 °C for 2 min, 40 cycles of 98 °C for 10 s, 50 °C for 15 s, and 68 °C for 1 min, and a final extension of 72 °C for 10 min. The complete mitochondrial genome was amplified by PCR using 12 pairs of primers (Table S5). The mitochondrial genome was sequenced and annotated following our previous study [4].

The complete mitogenome was obtained from *Bopyroides hippolytes* (GenBank accession number: MK905237). Because of the low quality of DNA extraction, only partial mitochondrial genomes were obtained from *Parabopyrella mortenseni* and *Bopyrella malensis*.

### Gene arrangement comparisons

PhyloSuite [35] was used to batch-download the 11 complete isopod mitochondrial genomes available from GenBank, and to assess genomic features and gene order. Phylograms and gene orders were visualized in iTOL [36]. For the purposes of visualization, we arbitrarily designated the beginning of the *cox1* gene as position 1 in each genome (pointing in the direction of *cox2*).

### Phylogenetic analyses

Most of the available sequence data for Bopyridae are mitochondrial genes and 18S rRNA. We constructed four datasets to assess phylogenetic relationships of bopyrids (Tables S2, S3, S4): (1) *cox1* dataset; (2) 18 s rRNA dataset; (3) combined *cox1* + 18 s rRNA dataset; (4) mitogenome dataset. Nucleotide sequences were used in the first three datasets and amino acid sequences were used in the mitogenome analyses. Missing sequences were treated as missing data. MAFFT [37, 38] was used to align sequences: nucleotide and amino acid sequences were aligned in batches (using codon and normal-alignment modes, respectively) with “–auto” strategy, whereas 18S rRNA gene was aligned using Q-INS-i algorithm, which takes secondary structure information into account. We used Gblocks v0.91b (http://molevol.cmima.csic.es/castresana/Gblocks_server.html) [39] to eliminate the ambiguous sequences after alignment, as they impact phylogenetic analyses [40]. Parameters were set as follows: type of sequence was set to codons for PCGs alignments; the minimum length of a block was set to 3 bp for PCGs and 2 bp for rRNA genes, and gap positions (−b5) were allowed with half. DNAsp v5 [41] and MEGA 5.03 [42] were used to calculate sequence composition and variability.

We tested the performance of homogeneous substitution models (using Maximum Likelihood (ML), and Bayesian Inference (BI)) for the single-gene dataset and COI-18S dataset. Data were partitioned by gene and codon position for the BI and ML analyses. PartitionFinder ver. 2.1.1 [43] was used to select partition schemes for BI, and ModelFinder [44] was used to select these for ML analysis in IQ-TREE, using the corrected Akaike Information Criterion (AICc). MrBayes ver. 3.2.6 [45] was used for BI, with four simultaneous runs with four chains each run for 10 million generations, sampling every 1000 trees. The first 25% of these trees were discarded as burn-in when computing the consensus tree (50% majority rule). Sufficient mixing of the chains was considered to be reached when the average standard deviation of split frequencies was below 0.01. ML analyses were conducted in IQ-TREE [46] with 1000 ultrafast BS [47].

Bayesian Inference with the CAT-GTR model based on mitogenome amino acid (AA) data outperform partitioned homogeneous models in isopods [35]. Consequently, we tested the performance of heterogeneous CAT-GTR model for mitogenome dataset, using AA sequence of 13 protein-coding genes (13PCGs). The CAT-GTR inference was implemented in PhyloBayes-MPI 1.7a on the beta version of the Cipres server [48], with default parameters (burnin = 500, invariable sites automatically removed from the alignment, two MCMC chains), and the analysis was stopped when the conditions indicated that a good run was reached (PhyloBayes manual: maxdiff < 0.1 and minimum effective size > 300).

## Supplementary Information

**Additional file 1: Table S1.** Partitioning strategies and best models from PartitionFinder and Morderfinder for the datasets.

**Additional file 2: Table S2.** Species and GenBank accession numbers (*cox1* gene) in the phylogenetic analyses.

**Additional file 3: Table S3.** Species and GenBank accession numbers (18S rRNA) in the phylogenetic analyses.

**Additional file 4: Table S4.** Species and GenBank accession numbers (mitochondrial genome) in the phylogenetic analyses.

**Additional file 5: Table S5.** Primers used for PCR amplification of the mitochondrial genome of *Bopyroides hippolytes.*

## Data Availability

All datasets are stored in NCBI. The accessions of the datasets are MW535162, MW535163, MK905237, MW540887, MW540885, MW540884. The analyzed data during this study are included in this published article and its supplementary information files.
