# Supplementary material for: Phylogenetic position of Bopyroides hippolytes, with comments on the rearrangement of the mitochondrial genome in isopods (Isopoda: Epicaridea: Bopyridae)
Source: BMC Genomics. 2022 Apr 2;23:253. doi: 10.1186/s12864-022-08513-9 (PMC8976331; doi:10.1186/s12864-022-08513-9)
Supplement: Supplementary file 1 — Additional file 1: Table S1. Partitioning strategies and best models from PartitionFinder and Morderfinder for the datasets. [file 12864_2022_8513_MOESM1_ESM.docx]

Table S1. Species and GenBank accession numbers (*cox1* gene) in the phylogenetic analyses

| Family | Subfamily | Taxa | Gene number |
| --- | --- | --- | --- |
| Bopyridae | Bopyrinae | *Probopyrus pandalicola* (Packard, 1879) | MK308333 |
|  |  | *Parabopyrella angulosa* (Bourdon, 1980) | MW535162 |
|  |  | *Bopyrella malensis* Bourdon, 1980 | MW535163 |
|  | Pseudioninae | *Gyge ovalis* (Shiino, 1939) | KY038053 |
|  |  | *Pseudione nephropsi* Shiino, 1951 | LC476591 |
|  |  | *Orthione griffenis* Markham, 2004 | KP412463 |
|  | Athelginae | *Athelges paguri* (Rathke, 1843) | KT209317 |
|  | Argeiinae | *Bopyroides hippolytes* (Kröyer, 1838)1 | MG319012 |
|  |  | *Bopyroides hippolytes* (Kröyer, 1838)2 | MK905237 |
|  |  | *Bopyroides hippolytes* (Kröyer, 1838)3 | MG314598 |
|  |  | *Argeia pugettensis* Dana, 1853 | MG316780 |
| Cymothoidae |  | *Cymothoa indica* Schioedte & Meinert, 1884 | MH396438 |
|  |  | *Asotana magnifica* Thatcher, 1988 | MK790137 |
