# Supplementary material for: Phylogenetic position of Bopyroides hippolytes, with comments on the rearrangement of the mitochondrial genome in isopods (Isopoda: Epicaridea: Bopyridae)
Source: BMC Genomics. 2022 Apr 2;23:253. doi: 10.1186/s12864-022-08513-9 (PMC8976331; doi:10.1186/s12864-022-08513-9)
Supplement: Supplementary file 2 — Additional file 2: Table S2. Species and GenBank accession numbers (cox1 gene) in the phylogenetic analyses. [file 12864_2022_8513_MOESM2_ESM.docx]

Table S2. Species and GenBank accession numbers (18S rRNA) in the phylogenetic analyses

| Family | Subfamily | Taxa | Gene number |
| --- | --- | --- | --- |
| Bopyridae | Bopyrinae | *Probopyrus pandalicola* (Packard, 1879) | EU848422 |
|  |  | *Probopyrus pacificiensis* Román-Contreras, 1993 | AF255683 |
|  |  | *Probopyrus buitendijki* (Horst, 1910) | KF765767 |
|  |  | *Parabopyrella angulosa* (Bourdon, 1980) | MW540887 |
|  |  | *Bopyrella malensis* Bourdon, 1980 | MW540885 |
|  | Keponinae | *Allokepon sinensis* (Danforth, 1972) | KF765766 |
|  | Pseudioninae | *Pseudione longicauda* Shiino, 1937 | KF765760 |
|  | Phyllodurinae | *Phyllodurus abdominalis* Stimpson, 1857 | KF765765 |
|  | Argeiinae | *Bopyroides hippolytes* (Kröyer, 1838) | MW540884 |
|  |  | *Argeia pugettensis* Dana, 1853 | KF765770 |
|  | Hemiarthrinae | *Hemiarthrus abdominalis* (Kröyer, 1840) | AF255684 |
|  | Athelginae | *Athelges takanoshimensis* Ishii, 1914 | KF765762 |
| Ionidae |  | *Ione cornuta* Bate, 1865 | MK765771 |
| Entoniscidae | Entioninae | *Portunion conformis* Muscatine, 1956 | KF765764 |
