# Supplementary material for: Phylogenetic position of Bopyroides hippolytes, with comments on the rearrangement of the mitochondrial genome in isopods (Isopoda: Epicaridea: Bopyridae)
Source: BMC Genomics. 2022 Apr 2;23:253. doi: 10.1186/s12864-022-08513-9 (PMC8976331; doi:10.1186/s12864-022-08513-9)
Supplement: Supplementary file 3 — Additional file 3: Table S3. Species and GenBank accession numbers (18S rRNA) in the phylogenetic analyses. [file 12864_2022_8513_MOESM3_ESM.docx]

Table S3. Species and GenBank accession numbers (complete and partial mitochondrial genome) in the phylogenetic analyses

| Order | Suborder | Taxa | Gene number |
| --- | --- | --- | --- |
| Mysida |  | *Neomysis japonica* Nakazawa, 1910 | NC_027510 |
| Amphipoda |  | *Metacrangonyx repens* (Stock & Rondé-Broekhuizen, 1986) | NC_019653 |
| Isopoda | Asellota | *Proasellus coiffaiti* Henry & Magniez, 1972 | LR536626 |
|  |  | *Proasellus meridianus* (Racovitza, 1919) | LR536625 |
|  |  | *Proasellus spelaeus* (Racovitza, 1922) | LR536624 |
|  |  | *Proasellus ortizi* Henry & Magniez, 1992 | LR536623 |
|  |  | *Proasellus rectus* Afonso, 1982 | LR536622 |
|  |  | *Proasellus coxalis* (Dollfus, 1892) | LR536621 |
|  |  | *Proasellus racovitzai* Henry & Magniez, 1972 | LR536620 |
|  |  | *Proasellus solanasi* Henry & Magniez, 1972 | LR536619 |
|  |  | *Proasellus parvulus* (Sket, 1960) | LR536618 |
|  |  | *Proasellus arthrodilus* (Braga, 1945) | LR536617 |
|  |  | *Proasellus assaforensis* Afonso, 1988 | LR536616 |
|  |  | *Proasellus cantabricus* Henry & Magniez, 1968 | LR536615 |
|  |  | *Proasellus cavaticus* (Leydig, 1871) | LR536614 |
|  |  | *Proasellus ebrensis* Henry & Magniez, 1992 | LR536613 |
|  |  | *Proasellus escolai* Henry & Magniez, 1982 | LR536612 |
|  |  | *Proasellus granadensis* Henry & Magniez, 2003 | LR536611 |
|  |  | *Proasellus hercegovinensis* (Karaman, 1933) | LR536610 |
|  |  | *Proasellus jaloniacus* Henry & Magniez, 1978 | LR536609 |
|  |  | *Bragasellus molinai* Henry & Magniez, 1988 | LR536608 |
|  |  | *Bragasellus peltatus* (Braga, 1944) | LR536607 |
|  |  | *Proasellus ibericus* (Braga, 1946) | LR536606 |
|  |  | *Proasellus karamani* Remy, 1934 | LR536605 |
|  |  | *Proasellus beticus* Henry & Magniez, 1992 | LR536604 |
|  |  | *Proasellus aragonensis* Henry & Magniez, 1992 | LR536603 |
|  |  | *Proasellus grafi* Henry & Magniez, 2003 | LR536602 |
|  |  | *Proasellus margalefi* Henry & Magniez, 1982 | LR536601 |
|  |  | *Asellus aquaticus* (Linnaeus, 1758) | GU130252 |
|  | Sphaeromatidea | *Sphaeroma serratum* (Fabricius, 1787) | GU130256 |
|  |  | *Sphaeroma terebrans* Bate, 1866 | MK460228 |
|  | Valvifera | *Glyptonotus* cf. *antarcticus* Eights, 1852 | GU130254 |
|  |  | *Idotea baltica* (Pallas, 1772) | DQ442915 |
|  | Phreatoicidea | *Eophreatoicus karrkkanj* | FJ790313 |
|  | Limnoriidea | *Limnoria quadripunctata* Holthuis, 1949 | KF704000 |
|  | Cymothoida | *Eurydice pulchra* Leach, 1815 | GU130253 |
|  |  | *Bathynomus* sp. | KU057374 |
|  |  | *Tachaea chinensis* Thielemann, 1910 | MF419232 |
|  |  | *Asotana magnifica* Thatcher, 1988 | MK790137 |
|  |  | *Ichthyoxenos japonensis* Richardson, 1913 | MF419233 |
|  |  | *Cymothoa indica* Schioedte & Meinert, 1884 | MH396438 |
|  | Epicaridea | *Argeia pugettensis* Dana,1853 | MG753775 |
|  |  | *Parabopyrella angulosa* (Bourdon, 1980) | MW553855; MW535162 |
|  |  | *Bopyrella malensis* Bourdon, 1980 | MW553856; MW535163 |
|  |  | *Gyge ovalis* (Shiino, 1939) | KY038053 |
|  |  | *Bopyroides hippolytes* (Kröyer, 1838) | MK905237 |
|  | Oniscidea | *Armadillidium vulgare* (Latreille, 1804) | GU130251 |
|  |  | *Ligia oceanica* (Linnaeus, 1767) | DQ442914 |
|  |  | *Mongoloniscus sinensis* (Dollfus, 1901) | MG709492 |
|  |  | *Armadillidium album* Dollfus, 1887 | KX289585 |
|  |  | *Armadillidium nasatum* Budde-Lund, 1885 | MF187611 |
|  |  | *Porcellionides pruinosus* (Brandt, 1833) | KX289584 |
|  |  | *Porcellio dilatatus petiti* Vandel, 1951 | KX289583 |
|  |  | *Porcellio dilatatus dilatatus* Brandt, 1833 | KX289582 |
|  |  | *Oniscus asellus* Linnaeus, 1758 | KX289581 |
|  |  | *Cylisticus convexus* (De Geer, 1778) | KR013002 |
|  |  | *Trachelipus rathkei* (Brandt, 1833) | KR013001 |
