# Supplementary material for: Phylogenetic position of Bopyroides hippolytes, with comments on the rearrangement of the mitochondrial genome in isopods (Isopoda: Epicaridea: Bopyridae)
Source: BMC Genomics. 2022 Apr 2;23:253. doi: 10.1186/s12864-022-08513-9 (PMC8976331; doi:10.1186/s12864-022-08513-9)
Supplement: Supplementary file 4 — Additional file 4: Table S4. Species and GenBank accession numbers (mitochondrial genome) in the phylogenetic analyses. [file 12864_2022_8513_MOESM4_ESM.docx]

Table S4 Partitioning schemes and best fit models identified by ModelFinder and PartitionFinder for the datasets

| Dataset | PartitionFinder | Moderfinder |
| --- | --- | --- |
| COI | Codon1: SYM+I+G  Codon2: GTR+G  Codon3: HKY+G | Codon1: GTR+F+I+G4  Codon2: TPM3+F+G4  Codon3: HKY+F+G4 |
| 18S | TIM2e+R3 | SYM+I+G |
| COI+18S | Codon1; 18S: SYM+I+G  Codon2: GTR+G  Codon3: HKY+G | 18S: TIM3e+G4  Codon1: TIM3e+I  Codon2: TPM3u+F+G4  Codon3: HKY+F+G4 |
