# Supplementary material for: Phylogenetic position of Bopyroides hippolytes, with comments on the rearrangement of the mitochondrial genome in isopods (Isopoda: Epicaridea: Bopyridae)
Source: BMC Genomics. 2022 Apr 2;23:253. doi: 10.1186/s12864-022-08513-9 (PMC8976331; doi:10.1186/s12864-022-08513-9)
Supplement: Supplementary file 5 — Additional file 5: Table S5. Primers used for PCR amplification of the mitochondrial genome of Bopyroides hippolytes. [file 12864_2022_8513_MOESM5_ESM.docx]

Table S5 Primers used for PCR amplification of the mitochondrial genome of *Bopyroides hippolytes*

| Fragment name | Gene or section | Primers | Primer sequence 5’-3’ | Length bp |
| --- | --- | --- | --- | --- |
| F1 | *16S*-*Cox2* | 45F1 | GTCCTTTTAGTGTATTTAAGC | 4238 |
|  |  | 45R1 | GGATAACAACACGATTATCG |  |
| F2 | *Cox2* | 45F2 | GTATTATACACACTAGATGAGG | 388 |
|  |  | 45R2 | CCACAACAATAGGCATGAATC |  |
| F3 | *Cox2*-*Atp6* | 45F3 | CTTGGACTGTACCTTCTTTGG | 778 |
|  |  | 45R3 | TGCCTAATCACAGAGGTATTG |  |
| F4 | *Atp6* | 45F4 | GCTACCACTTCTTAGCTGTTC | 345 |
|  |  | 45R4 | CAATCCCTGAGGTGACTAGAG |  |
| F5 | *Atp6*-*Nad1* | 45F5 | CCAGGGGGACTCCAGTAGC | 2017 |
|  |  | 45R5 | GCCAACAGGGGCAGGCAGATG |  |
| F6 | *Nad1* | 45F6 | TGTGCCTCCTCTGTATTCTG | 581 |
|  |  | 45R6 | GTAAAGTTTTAGGCTACACAC |  |
| F7 | *Nad1*-*12S* | 45F7 | GACAAAGAAGGACCGGCTAT | 656 |
|  |  | 45R7 | CTACTTTAAGCCTAGAATTC |  |
| F8 | *12S* | 45F8 | GGAAAAGAGCAGTGCCAGCAC | 564 |
|  |  | 45R8 | CTTATCTCGTAGTCCGAGAGTG |  |
| F9 | *12S*-*Cytb* | 45F9 | GCATTGAGAGTATTCAACCG | 1709 |
|  |  | 45R9 | TATCAATTCTAGTCCTACTT |  |
| F10 | *Cytb* | 45F10 | CAAACTTGCTAGGAATTGCTCG | 492 |
|  |  | 45R10 | CAACTGCATTCTTAGGGTATG |  |
| F11 | *Cytb* -*16S* | 45F11 | AGGTAGGATTATCTACTGAG | 4942 |
|  |  | 45R11 | CTTAGAGACCATATCAAAG |  |
| F12 | *16S* | 45F12 | GATAGAAACCAACCTGGCTC | 475 |
|  |  | 45R12 | GTATCCTAACTGTGCAAAGG |  |
